# Supplementary material for: Fungal X-Intrinsic Protein Aquaporin from Trichoderma atroviride: Structural and Functional Considerations
Source: Biomolecules. 2021 Feb 23;11(2):338. doi: 10.3390/biom11020338 (PMC7927018; doi:10.3390/biom11020338)

Figure S10. Light microscopy of hyphae and spores of the *Trichoderma atroviride* wild strain and the five Δ*Triat*XIP mutants. The scale bar represents 20 µm. Samples were coloured with toluidine blue (1%) for 1 min prior to the observations. ChlSp, Chlamydospores; Sp, spores; Hy, hyphes.

***Tichoderma atroviride* WT**

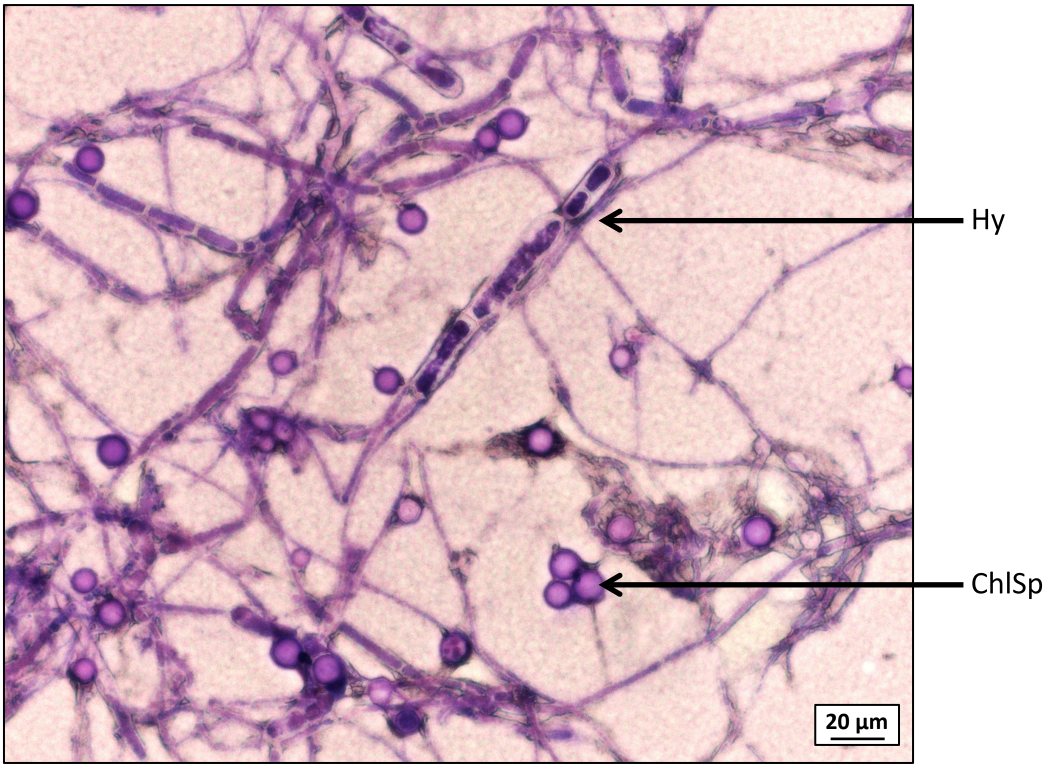

Δ*Triat*XIPa

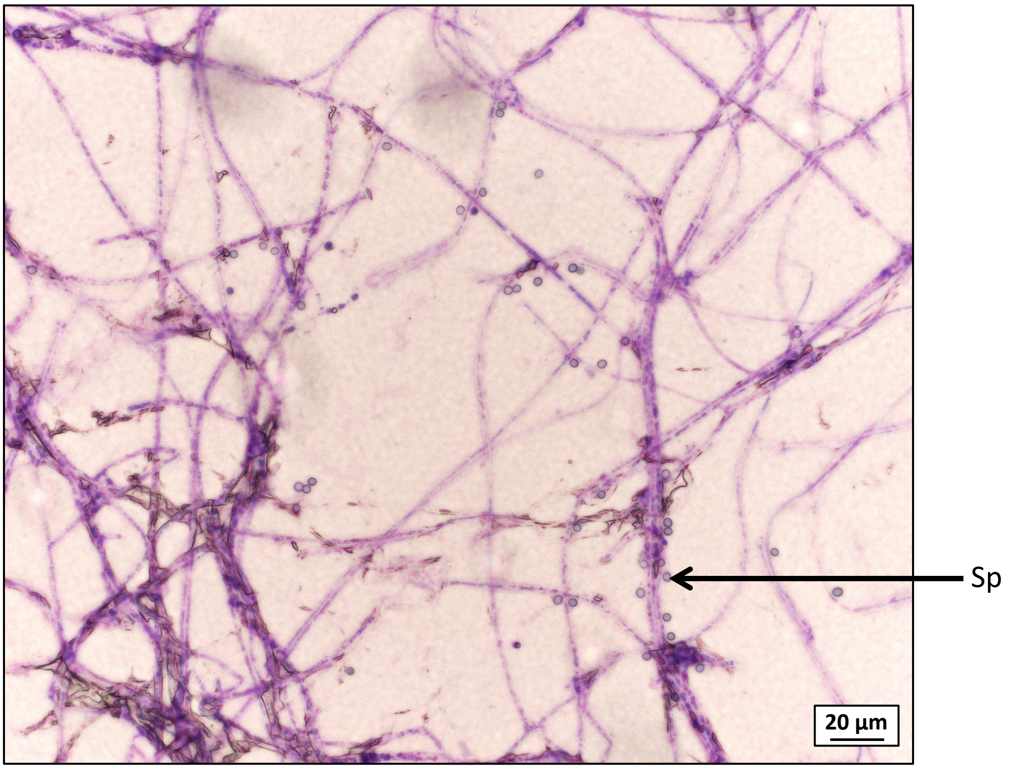

ΔTriatXIPb

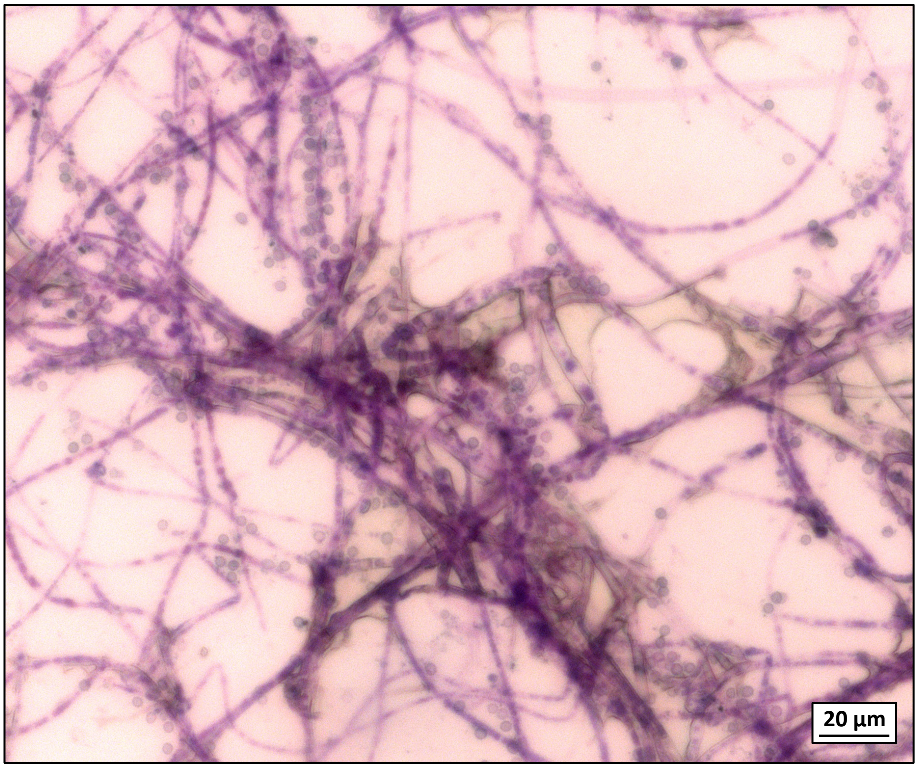

ΔTriatXIPc

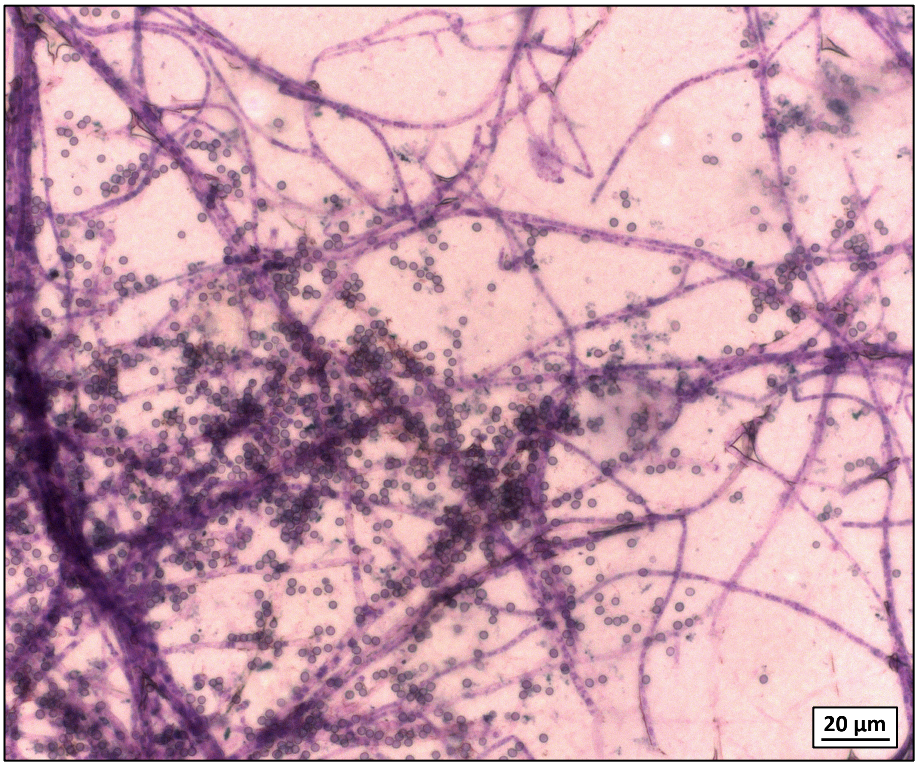

Δ*Triat*XIPd

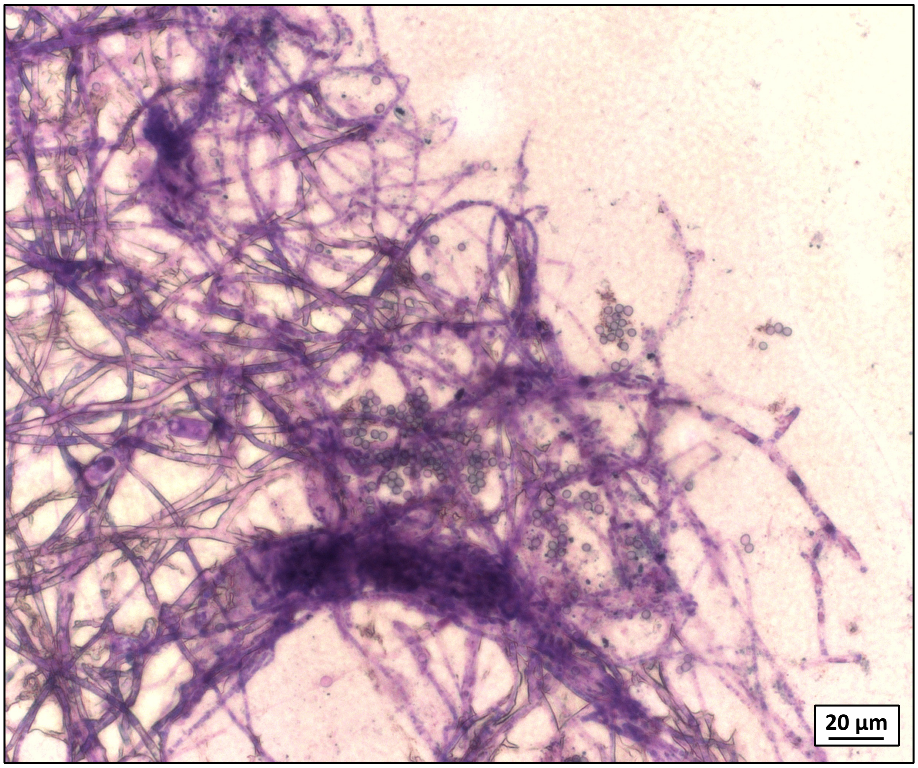

Δ*Triat*XIPe

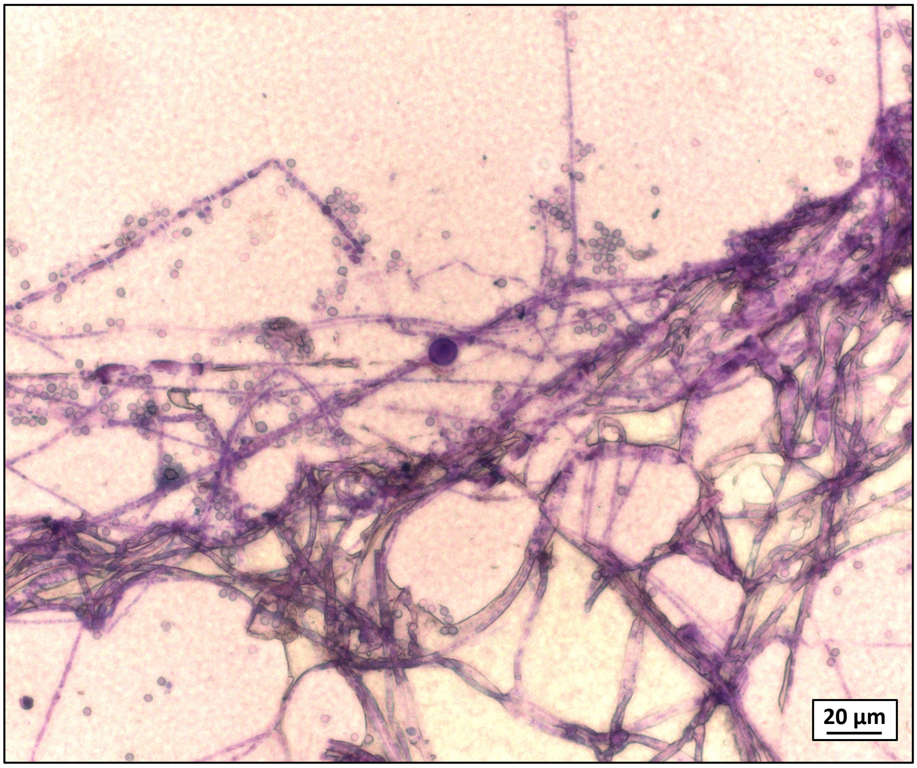

Supplement: Supplementary file 1 [file biomolecules-11-00338-s001.zip › Figures Sup PDF/FigS10_Microscopic_slides.pdf]
