# Supplementary material for: Fungal X-Intrinsic Protein Aquaporin from Trichoderma atroviride: Structural and Functional Considerations
Source: Biomolecules. 2021 Feb 23;11(2):338. doi: 10.3390/biom11020338 (PMC7927018; doi:10.3390/biom11020338)

Figure S11. Confrontations of the five Δ*Triat*XIP mutants and the wild strain with *Rhizoctonia solani*, *Botrytis cinerea* and *Fusarium graminearum* on PDA medium.

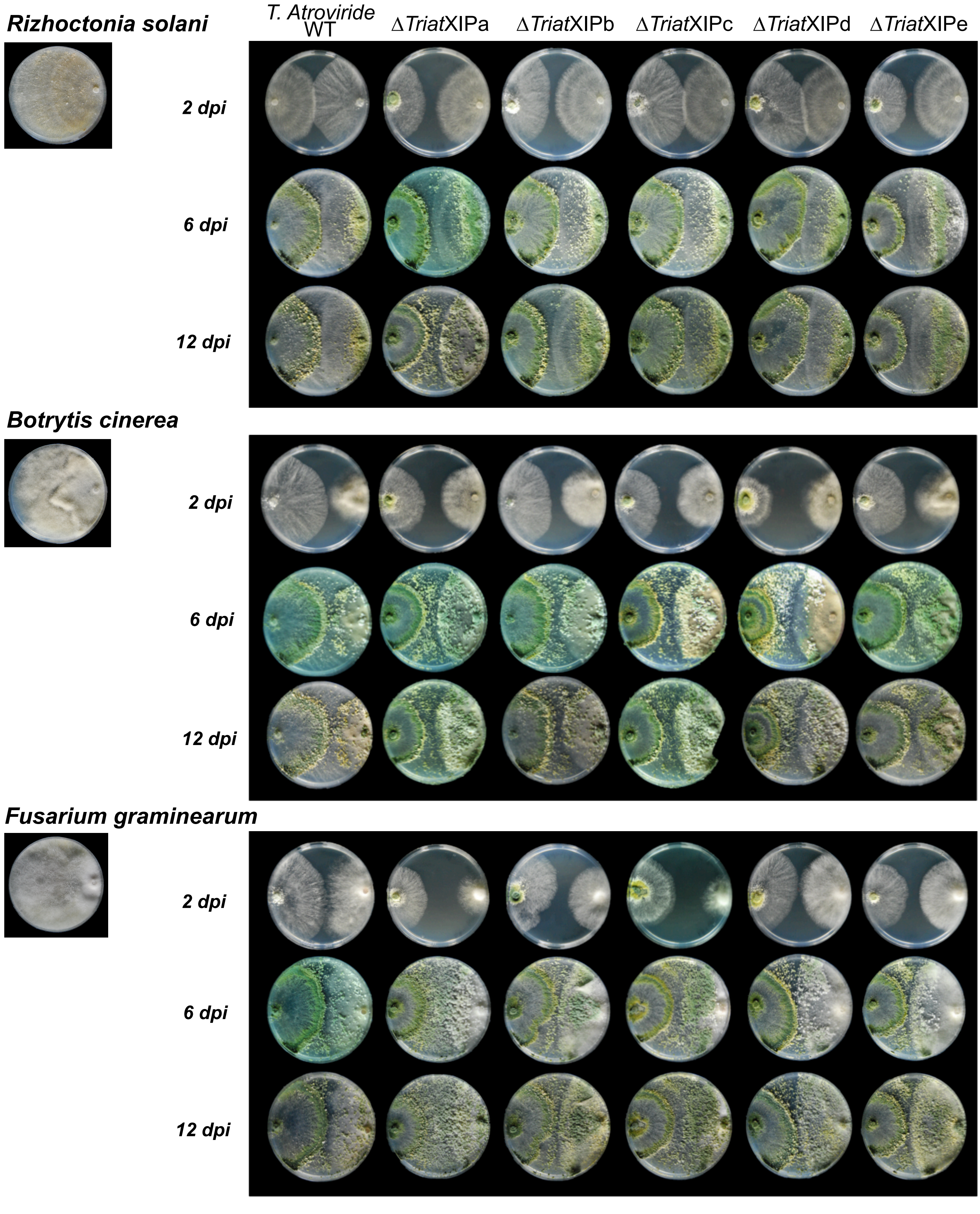

Supplement: Supplementary file 1 [file biomolecules-11-00338-s001.zip › Figures Sup PDF/FigS11_Mycoparasitism_microscopy.pdf]
