# Supplementary material for: Fungal X-Intrinsic Protein Aquaporin from Trichoderma atroviride: Structural and Functional Considerations
Source: Biomolecules. 2021 Feb 23;11(2):338. doi: 10.3390/biom11020338 (PMC7927018; doi:10.3390/biom11020338)
Supplement: Supplementary file 1 [file biomolecules-11-00338-s001.zip › Figures Sup PDF/FigS12_list_of_BBiolog_metabolites.pdf]

Figure S12. **List of the 95 metabolites used for the study.** These metabolites are available with the Phenotype MicroArray™ (PM) system (Biolog FF MicroPlate, MT2 serie). For data analyses, the metabolites were classified into 8 biochemical classes.

Medium Class	Class size	Class list
amines&amides	5	D-Glucosamine, Glucuronamide, Alaninamide, 2-Amino Ethanol, Putrescine
amino-acids	16	Succinamic Acid, Succinic Acid, Succinic Acid Mono-Methyl Ester, N-Acetly-L-glutamic Acid, L-Alanine, L-Alanyl-Glycine, L-Asparagine, L-Aspartic Acid, L-Glutamic Acid, Glycyl-L-Glutamic Acid, L-Ornithine, L-Phenylalanine, L-Proline, L-Pyroglutamic Acid, L-Serine, L-Threonine
carboxylic acids	18	D-Galacturonic Acid, D-Gluconic Acid, D-Glucuronic Acid, 2-Keto-D-Gluconic Acid, gamma-Amino-butyric Acid, Bromosuccinic Acid, Fumaric Acid, beta-Hydroxy-butyric Acid, gamma-Hydroxy-butyric Acid, p-Hydroxyphenylacetic Acid, alfa-Keto-glutaric Acid, D-Lactic Acid Methyl Ester, L-Lactic Acid, D-Malic Acid, L-Malic Acid, Quinic Acid, D-Saccharic Acid, Sebacic Acid
miscellaneous	1	tween80
nucleic acids	3	Adenosine, Uridine, Adenosine-5-Monophosphate
polyols	9	Adonitol, D-Arabitol, i-Erythritol, Glycerol, m-Inositol, Maltitol, D-mannitol, D-Sorbitol, Xylitol
polyoses	21	Amygdalin, Arbutin, D-Cellobiose, alfa-Cyclodextrin, beta-Cyclodextrin, Dextrin, Gentiobiose, Glycogen, alfa-D-Lactose, Lactulose, Maltose, Maltotriose, D-Melezitose, D-Melibiose, Palatinose, D-Raffinose, Salicin, Stachyose, Sucrose, D-Trehalose, Turanose
simple oses	22	N-Acetyl-D-Galactosamine, N-Acetyl-D-Glucosamine, N-Acetyl-D-Mannosamine, D-Arabinose, L-Arabinose, D-Fructose, L-Fucose, D-Galactose, alfa-D-Glucose, Glucose-1-Phosphate, D-Mannose, alfa-Methyl-D-Galactoside, beta-Methyl-D-Galactoside, alfa-Methyl-D-Glucoside, beta-Methyl-D-Glucoside, D-Psicose, L-Rhamnose, D-Ribose, Sedoheptulosan, L-Sorbose, D-Tagatose, D-Xylose
