# Supplementary material for: Fungal X-Intrinsic Protein Aquaporin from Trichoderma atroviride: Structural and Functional Considerations
Source: Biomolecules. 2021 Feb 23;11(2):338. doi: 10.3390/biom11020338 (PMC7927018; doi:10.3390/biom11020338)

Figure S13. Colinearity between biomass production (*i.e* mycelial growth) and respiration (*i.e.* catabolism) per biochemical class from the *T. atroviride* wild strain and the five Δ*Triat*XIP mutants in cultivation on the 95 metabolites using the Phenotype MicroArray™ (PM) system (Biolog FF MicroPlate, MT2 serie).

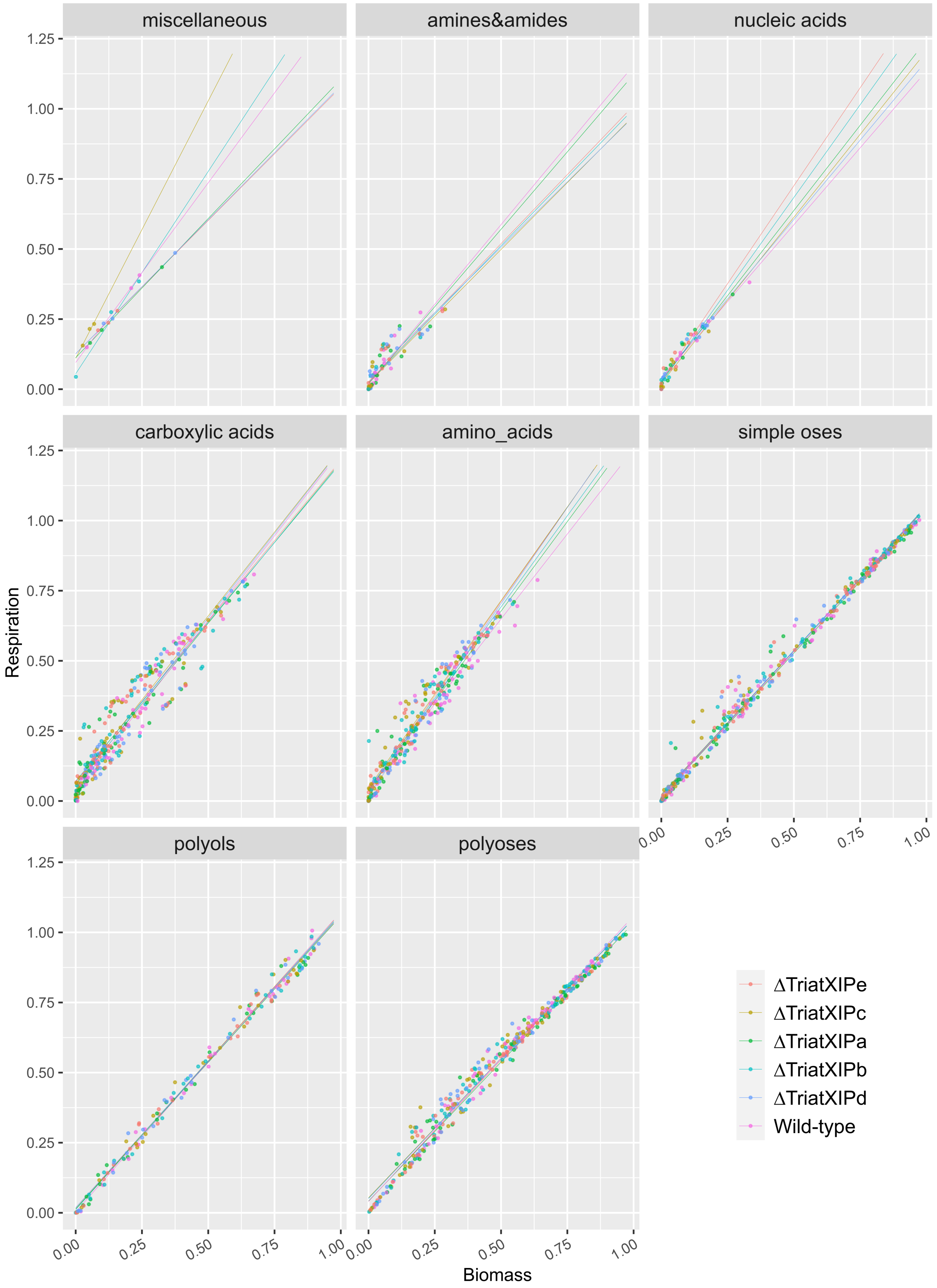

Supplement: Supplementary file 1 [file biomolecules-11-00338-s001.zip › Figures Sup PDF/FigS13_Colinearity_per biochemical_class.pdf]
