# Supplementary material for: Fungal X-Intrinsic Protein Aquaporin from Trichoderma atroviride: Structural and Functional Considerations
Source: Biomolecules. 2021 Feb 23;11(2):338. doi: 10.3390/biom11020338 (PMC7927018; doi:10.3390/biom11020338)
Supplement: Supplementary file 1 [file biomolecules-11-00338-s001.zip › Figures Sup PDF/FigS15_PValues.pdf]

Figure S15. Comparative carbon source utilization profiles of the five ∆*Triat*XIP mutants and the *T. atroviride* parental strain. The order of the organic sources is the rank of respiration rate on 95 organic sources and water after 96h of incubation. The mycelial respiration corresponds to the mean of three independent biological experiments per strain. In the green sidebar, respiration rates are specified from the five ∆*Triat*XIP that were significantly different from that of the *T. atroviride* parental strain, as verified by T-Test ($p<0.05$), are specified. In the red sidebar, the data are specified that were not significantly different ($p>0,05$) between the five ∆*Triat*XIP mutants and the parental strain, but which reflect interesting metabolic trends are specified.

Medium	Medium Class	vs	*p*value
D-Tagatose	simple oses	mutant/wild-type	0.000
L-Rhamnose	simple oses	mutant/wild-type	0.000
L-Phenylalanine	amino-acids	mutant/wild-type	0.000
D-Mannose	simple oses	mutant/wild-type	0.000
L-Alanyl-Glycine	amino-acids	mutant/wild-type	0.005
Glycyl-L-Glutamic Acid	amino-acids	mutant/wild-type	0.006
D-Ribose	simple oses	mutant/wild-type	0.007
L-Aspartic Acid	amino-acids	mutant/wild-type	0.013
beta-Methyl-D-Galactoside	simple oses	mutant/wild-type	0.013
N-Acetyl-D-Glucosamine	simple oses	mutant/wild-type	0.013
gamma-Amino-butyric Acid	carboxylic acids	mutant/wild-type	0.014
Glycerol	polyols	mutant/wild-type	0.015
L-Arabinose	simple oses	mutant/wild-type	0.030
L-Asparagine	amino-acids	mutant/wild-type	0.032
L-Malic Acid	carboxylic acids	mutant/wild-type	0.043
gamma-Hydroxy-butyric Acid	carboxylic acids	mutant/wild-type	0.043
D-Cellobiose	polyoses	mutant/wild-type	0.061
L-Threonine	amino-acids	mutant/wild-type	0.066
Fumaric Acid	carboxylic acids	mutant/wild-type	0.075
L-Ornithine	amino-acids	mutant/wild-type	0.078
Sebacic Acid	carboxylic acids	mutant/wild-type	0.079
D-Melibiose	polyoses	mutant/wild-type	0.087
N-Acetly-L-glutamic Acid	amino-acids	mutant/wild-type	0.091
p-Hydroxyphenylacetic Acid	carboxylic acids	mutant/wild-type	0.095
D-Psicose	simple oses	mutant/wild-type	0.108
Maltitol	polyols	mutant/wild-type	0.109
Arbutin	polyoses	mutant/wild-type	0.121
Xylitol	polyols	mutant/wild-type	0.151
D-Trehalose	polyoses	mutant/wild-type	0.166
Turanose	polyoses	mutant/wild-type	0.182
Adenosine-5-Monophosphate	nucleic acids	mutant/wild-type	0.193
alfa-D-Glucose	simple oses	mutant/wild-type	0.207
Salicin	polyoses	mutant/wild-type	0.208
D-Glucuronic Acid	carboxylic acids	mutant/wild-type	0.209
L-Alanine	amino-acids	mutant/wild-type	0.216
L-Sorbose	simple oses	mutant/wild-type	0.252
Stachyose	polyoses	mutant/wild-type	0.262
alfa-Keto-glutaric Acid	carboxylic acids	mutant/wild-type	0.263
Alaninamide	amines&amides	mutant/wild-type	0.270
m-Inositol	polyols	mutant/wild-type	0.283
L-Proline	amino-acids	mutant/wild-type	0.295
Palatinose	polyoses	mutant/wild-type	0.297
Succinic Acid Mono-Methyl Ester	amino-acids	mutant/wild-type	0.338
Adonitol	polyols	mutant/wild-type	0.338
Succinic Acid	amino-acids	mutant/wild-type	0.360
Adenosine	nucleic acids	mutant/wild-type	0.381
L-Glutamic Acid	amino-acids	mutant/wild-type	0.387
D-Arabitol	polyols	mutant/wild-type	0.398
alfa-Methyl-D-Glucoside	simple oses	mutant/wild-type	0.416
Amygdalin	polyoses	mutant/wild-type	0.416
Bromosuccinic Acid	carboxylic acids	mutant/wild-type	0.425
D-Saccharic Acid	carboxylic acids	mutant/wild-type	0.428
D-Fructose	simple oses	mutant/wild-type	0.430

Supplemental Figure S16 - next

Medium	Medium Class	vs	*p*value
L-Lactic Acid	carboxylic acids	mutant/wild-type	0.439
alfa-Cyclodextrin	polyoses	mutant/wild-type	0.443
Sucrose	polyoses	mutant/wild-type	0.472
D-Arabinose	simple oses	mutant/wild-type	0.477
Succinamic Acid	amino-acids	mutant/wild-type	0.480
N-Acetyl-D-Galactosamine	simple oses	mutant/wild-type	0.488
D-Mannitol	polyols	mutant/wild-type	0.488
Uridine	nucleic acids	mutant/wild-type	0.495
alfa-Methyl-D-Galactoside	simple oses	mutant/wild-type	0.514
i-Erythritol	polyols	mutant/wild-type	0.523
2-Amino Ethanol	amines&amides	mutant/wild-type	0.529
alfa-D-Lactose	polyoses	mutant/wild-type	0.535
Sedoheptulosan	simple oses	mutant/wild-type	0.536
Glucose-1-Phosphate	simple oses	mutant/wild-type	0.538
D-Melezitose	polyoses	mutant/wild-type	0.577
L-Fucose	simple oses	mutant/wild-type	0.582
tween80	miscellaneous	mutant/wild-type	0.594
beta-Methyl-D-Glucoside	simple oses	mutant/wild-type	0.612
D-Malic Acid	carboxylic acids	mutant/wild-type	0.621
Dextrin	polyoses	mutant/wild-type	0.651
N-Acetyl-D-Mannosamine	simple oses	mutant/wild-type	0.664
D-Lactic Acid Methyl Ester	carboxylic acids	mutant/wild-type	0.665
Gentiobiose	polyoses	mutant/wild-type	0.674
D-Sorbitol	polyols	mutant/wild-type	0.677
Maltotriose	polyoses	mutant/wild-type	0.684
D-Raffinose	polyoses	mutant/wild-type	0.704
L-Pyroglutamic Acid	amino-acids	mutant/wild-type	0.713
Maltose	polyoses	mutant/wild-type	0.716
Glucuronamide	amines&amides	mutant/wild-type	0.723
D-Glucosamine	amines&amides	mutant/wild-type	0.766
Putrescine	amines&amides	mutant/wild-type	0.778
D-Gluconic Acid	carboxylic acids	mutant/wild-type	0.793
D-Xylose	simple oses	mutant/wild-type	0.835
Quinic Acid	carboxylic acids	mutant/wild-type	0.853
beta-Hydroxy-butyric Acid	carboxylic acids	mutant/wild-type	0.858
beta-Cyclodextrin	polyoses	mutant/wild-type	0.861
L-Serine	amino-acids	mutant/wild-type	0.865
2-Keto-D-Gluconic Acid	carboxylic acids	mutant/wild-type	0.868
Lactulose	polyoses	mutant/wild-type	0.885
D-Galactose	simple oses	mutant/wild-type	0.895
D-Galacturonic Acid	carboxylic acids	mutant/wild-type	0.915
Glycogen	polyoses	mutant/wild-type	1.000
