# Supplementary material for: Fungal X-Intrinsic Protein Aquaporin from Trichoderma atroviride: Structural and Functional Considerations
Source: Biomolecules. 2021 Feb 23;11(2):338. doi: 10.3390/biom11020338 (PMC7927018; doi:10.3390/biom11020338)
Supplement: Supplementary file 1 [file biomolecules-11-00338-s001.zip › Figures Sup PDF/FigS16_qPCR_detailed.pdf]

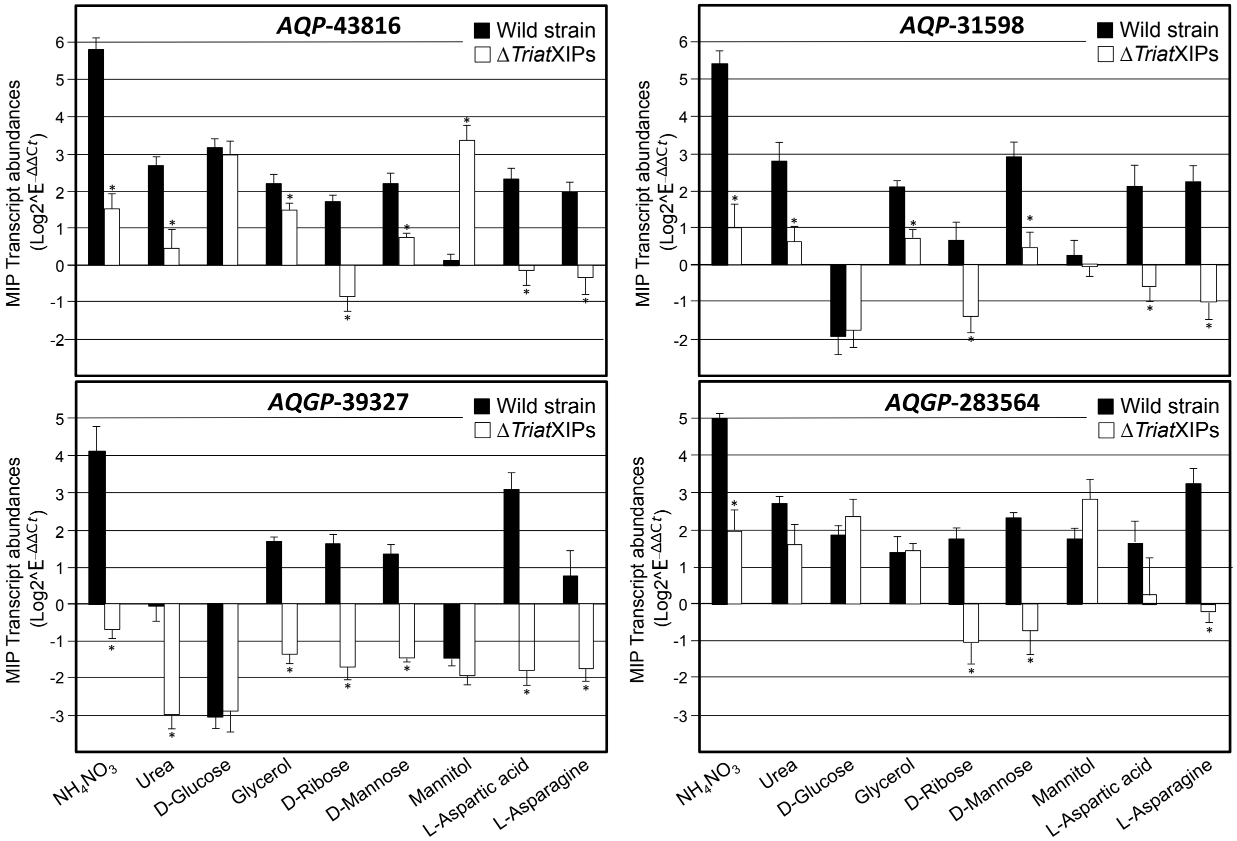

Figure S16. Detail of the relative transcription ratios of the expressed MIP genes from *Trichoderma atroviride* wild type and the five Δ*Triat*XIP mutants upon growth on PDA medium supplemented with various inorganic and organic compounds presented in Figure 6b. Differential transcript levels for each gene were estimated using real-time qRT-PCR analyses and normalized by the expression of five housekeeping genes. Relative transcript abundance rates were obtained by the $E^{-\Delta\Delta Ct}$ method. Data correspond to the means of three biological replicates for the wild strain and the mean of the five Δ*Triat*XIP mutants. Bars represent the biological standard error. *, Data are statistically significantly different between the wild and the five Δ*Triat*XIP strains, as verified by one-way ANOVA analysis followed by Tukey's post hoc test (*p<0.05).
