# Supplementary material for: Fungal X-Intrinsic Protein Aquaporin from Trichoderma atroviride: Structural and Functional Considerations
Source: Biomolecules. 2021 Feb 23;11(2):338. doi: 10.3390/biom11020338 (PMC7927018; doi:10.3390/biom11020338)
Supplement: Supplementary file 1 [file biomolecules-11-00338-s001.zip › Figures Sup PDF/FigS1_MIP_Trichoderma.pdf]

Figure S1. Protein MIP sequences from *Trichoderma* genus and XIPs from *Aspergillus_terreus* and *Aspergillus_terreus*. The protein IDs are mentioned. The protein sequences were extracted from JGI or NCBI portals.

>Trichoderma_atroviride_319992 (*Triat*XIP)
MDLAAFDGSFAPLVRPQAVRLTPWYRRRDYFVGQWFEPALWRSAVVELIATCCQVFVSGQIVATISTYGTPQLGA
YIGISNLVLIATFIYAVAPASGGHMNPTITFAAVLTGLCSVPRGLLYLVGQTAGGALGGGILLGIWGKERAIAVR
GGGCWYDPSQANPGQIYLNETFASFVLLFLAFGVGLDPRQAALFGPRMGPALVGASLGLVSFSTSGIIPGYAGAQ
MNPAKCFGNGIARMDLSYQWIYWFGPAVAGIMMGILYNLIPPHHAELCKRKSREMSREMTSDSMAERAEASVIAS
A*
>Trichoderma_atroviride_283564 (*Triat*Fps-like)
MCHEAIGGSLWEDRLPHAGIDETISSSRQNVRGSQEDTERGSLRRRSRTSRRSTFRSVAPLTTAGQGTQFNLAGP
SESQQLRSAHEPFVHPGYGDLNPSYEQPNNTKPIWSLAKPLPRVVRPGMVPTKNELLESCINAELPAENSQKLGL
DVDPNELEKGRIDKSADVRKMAAQVTDARVQRENNFIKTILASDAESASGDVAQLVQTRSSQRRATITRPPVQSP
LDTVEESVSDHLSTASKKRRSRESQRLDEAEGEIDLGPDELIEGNRSLETLRIDQEAYPEDLHPLVQNLVEDEIH
NNHTVWSVVRTHHREALAESLAVFIQLTVGFCADIAVTVANAGNPNTTAWAWGFASMMGIYISGGVSGAHLNPTI
TVMLWFFRGFPKRKMPEYFLAQFIGAFCACFAAYGVYYVSIQHYVSSGTGTDQEIMNCFVTNQRNPYINVPTALF
TEFVGTMCLTVVVLALGDDQNAPPGAGMNSLIIGLVIVCLTISFANQTGAALNPSRDFGPRLALLALGYGSELFT
NPYWFYGPFAGTLMGSFTGAFLYDFFIFTGGESPVNYPLDRTQRALRKSGMKWRRRLHLTPKEEEDKVV*
>Trichoderma_atroviride_39327 (*Triat*Fps-like)
MSPSLEEEQLAWSRIREYCMDAFSEFFGTMVLILFGDGVVAQVVLSGSTKGDYQSISWGWGIAVMFGVFVGGKSG
GHLNPAVTFANCLYRGHPWRKLPVYALAQLLGAMVGAAIVYGNYKSAFDAFEGGAGIRTVTGPTASAGVFCTYPA
PFMTRTGMFFSEFIASSILMFCIFALVDPNNVNAGHMMPLALFFLIFGIGACFGWETGYAINLARDFGPRLVSYM
IGYGHEVWSAGGYYFWIPMVAPFCGTAFGGFLYDVFLYTGNSPINTPMLGLTRLFRPRKNVWSNTRPSAIDTKV*
>Trichoderma_atroviride_90169 (*Triat*Other-AQGP)
MTTRELNRLHTHDLELGTTEATQKHVPRNVVPPRRISQRRLDFEHRRPRWLRECIAEATGVFMYVLPGIGAIATF
TLNAASPIGASAFGSLFAIGFAFALGIAFAIITCAPTSGGHFSPAVTICLCIWQGFPLKKVPYYIFSQLVGGFLA
ALVLMGIYHQQINEMKELLLAAGKPLVANGAPASILCSFPNPDQSMGYVFFTEFICDCFVGFIIWAAIDPANPFI
SPAVAPLMIGLAYAAMAWGFGDVTLTMNMARDLGPRIVAAIFFGKEAFSYKNYSPIAILVSIPATMVSSAFYEFV
FRDSVSVIQSGHAVHEDGDEALVRHITRTTTVEGIDERRGDYKS*
>Trichoderma_atroviride_6990 (*Triat*AQP)
MRRPSASRDTVRNELVVVFGEFYGTFMFLLMSYIGTQAAIDNNSPGNPEAPLFPFSLMYIAASFGTALAVNVWVF
YRVTGGLFNPAVTLGLVLVRAITPLRGLLVFPTQIVAGIAAAAVTDALLPGPLLVANKLSSGTSISRGLFIEMFL
TAQLVITVYFLAVEKHRATFLAPLGIGLAVFIAHICGTNFTGTGINPARSFGPCVVTSFTGYQWIYWAGPFMGAL
LAFAVYSILKWLEYHNANPGQDDDSNVKRNPAGFAITSNDRQYSSSTTGSKPHTPTGHNPRDSGIAQTNSAPQGF
QAV*
>Trichoderma_atroviride_31598 (*Triat*AQP)
MDSLRIFKQRSVSGDNNRNRDRFHPSTIQKHLIASAGEFVGTFFFLWFAYAGSMQYIKQATLSPPSGGISDTTVF
FIAHVYSFSLLVNVWAFYRISGGLFNPAVTLGMCLAGTLPWVRAAFLVPAQIIASMCAGGLARCMFPGDLAVANS
VLSSDTSIVRGLFIEMFFTAFLVFVVLMLSAERSKDTFIAPIGIGLALFVAMLAGTSYTGASLNPVRSFGCAVAT
PSFPGYEWIYWLGPFMGALVAAGFYRFVKLSHYEEANPNREETDHPTGDQP*
>Trichoderma_atroviride_43816 (*Triat*AQP)
MPANRIHTLSKRYEKALVIGVGEFCGTFMFLLLSFMGAQVALENNPAADGHKLEPATLLYIASSFGTALAVNVWV
FYRVTGGMFNPAVTLGLVLVGAVKPLRALIIILPTQIIAGIAAAATVSALLPGHLDVTNSLGGGTSTAQGLFIEMF
LTAQLVLTVYFLAVEKHRATFLAPVGIGVSVFIAHLAGTNFTGTGINPVRSLGPAVVTGRFEGYHWIYWLGPCLG
ALLSFAVYSLLKALEYQIANPGQDAGDPETANEEALAVEEMGTEGLAIVRSRTRSGSAASEARYKRDVAMRRGSA
VDGSAVSAADAV*

>Trichoderma_harzianum_488926
MDLAAFDGSFAPGVRPGAVRLTPWYRRRDYFVGQWVDISVWKSAVVEFVATSCLVFLSGQITATLESYQTPQVGG
YIGISNIILIATFIYATAPASGGHLNPMITFSAILTGLCSVPRGILYMCAQTLGGALAGGILLGVWGPKRATSLQ
GGGCWYDPSQANPGQIYLNEVFASFVLLFLSFGVGLDPRQAALFGPRMGPLLVGASLGLVSFATSGIIPGYAGAQ
MNPSRCLAFGIARRDMTYQWVWWFGPAVGCLMMGIFYNLIPPHHVELSKQKSKESRMNSMAGNTEVPEV*
>Trichoderma_harzianum_90014
MELLSPPLEEEQLAWSKIRSYCQDFFSEFLGTMTLILFGDGVVAQVVLSGGTKGDYQSISWGWGIAVMLGVYVGG
KSGGHLNPAVTFANCLYRGHPWRKLPVYALAQLLGAMTGAAIVYANYKSAFDAFEGGAGIRTVTGPTATAGVFCT
YPAAFMTRTGMFFSEFIASSILMFCIFALADPNNIGAGNLMPLCLFFLIFGIGACFGWETGYAINLARDFGPRLV
SFMIGYGHEVWSAGGYYFWIPMVAPFCGCAFGGFLYDVFIFTGNSPINTPMLGLQRLMRPRKSVWSNTHPAAIEN
KV*
>Trichoderma_harzianum_490850

MREPFSEFFGVLILVLFGDGSVAQVVLGKGAKGDWNNINWGWALGVMLGVYCGGVSGAHLNPAVTLANCIFRKFP
WKKLPVYALAQLLGAMAASLIVYGNYKSAIDVFEGGQGIRTVGLDTSTAGIFCTYPAPFLTKTGQFFDECIGSSI
LMFCLYALLDDGNIGAGNLTPLGLFFVIYGIGACFGSNTGYAINPARDLGPRIMSHAVGYGHQVWTAGDYYFWVP
VIAPFLGCTFGGFLYDTFIYTGDSPINAPYMGFTRFMGVHAKARRTAMV*
>Trichoderma_harzianum_82211
MTSSRRVLNETREDQDRSSLRRRSRASRRSTFRSVAPITTAGQGTQFNLAGPTDTPQLRTAHEPFVHPGYSDLNP
SYEQPNNAKPIWSLAKPLPRVVRPGMVPTKNELLENCVNAELPAENSQNLGLDVDPNEIEKGRIEKSADLRKMGA
QVTDARQQRENNFIKTILAADAEAQSDGLPQLVKTRSSQRRATITRPSIQSPLYTVQEGLSEHTSERHKDSHESQ
RSGQGEGEFDPGPDELIEGNRSLETLRLDQDAYPEDLHPLVQDLVEEEIHNNHTIWSVIRTHHREALAESLAVFV
QLTIGFCADLSVTVAKAGNPNTTDWAWGFATMIGIYISGGVSGAHLNPTITIMLWFFRGFPKRKMPEYFLAQFLG
AFCACFAAYGVYYVSIKHYLLTGVDDDIINCFVTSQRSSYINAPTALFNEFIGTMCLTIVVLALGDDQNAPPGAG
MNSLIIGLIITCLSMSFANQTGAALNPSRDFGPRLALLALGYTSELFTNPYWFYGPWAGTLLGSFMGAFLYDFFI
FTGGESPINYPLERTQRAFHKSGMKWRRRLHLTPKQEEERIV*
>Trichoderma_harzianum_92358
MSSPTEQLNRLHTHDLELGTTDAVQKHISRNVVPPRRVSQRRLDFEHRRPRWLRECIAEATGVFMYVLPGIGAIT
SFTINATNPIGSTAFGSLFSIGFAFALGIAFAIITCAPTSGGHFSPAVTIALWFWQGFPLKKVPYYIFSQLLGGF
IAALVLMGIYHQQLDEMKQVLLAAGEPLVANGAPASVLCSFPNPGQSMGYVFMTEFFCDCFVGLIIWACLDPANP
FVSPSLAPLVIGLAYGAMAWGFGANTLTMNMARDFGPRVVAAIFYGREAFSYMNYAAIGIFTSIPATLVSSAFYE
FVMRDSLSVIGTGHAVHADGDEALVRHITRTTTVDGIEERRGEYKS*
>Trichoderma_harzianum_99286
MASSGIFKQHSGSGDNGRSRDGFHPSTIQKHLIASAGEFVGTFFFLWFAYAGSMQYVKQATLSPLSGGISDTTVF
FIAHVYSFSLLVNVWAFYRISGGLFNPAVTLGMCLAGTLPWVRAAFLVPAQIIASMCAGGLARCMFPGDLAVANS
VLSSDTSIVRGLFIEMFFTAFLVFVVLMLAAERSKDTFIAPIGIGLALFVAMLAGTSYTGASLNPVRSFGCAVAT
PSFPGYEWIYWLGPFMGAVVAAGFYRFIKWSRYEEVNPNREETDHPNGDQPQPAQHSSV*
>Trichoderma_harzianum_98742
MHRQYAPPREPIRNEIVVVFGEFCGTFMFLLMSFIGTQAALDNNDPTNPNAPLFPFSLLYVASSFGAALAVNVWV
FYRVTGGMFNPAVTLGLVLVGAVKPIRGLLIFPAQIVAGIAAAAVTDALLPGPLLVANKLASGTSISRGLFIEMF
LTAQLVITVYFLAVEKHRATFLAPLGIGLAVFIAHICGTNFTGTGINPARSFGPAVVTDFTGYQWIYWVGPLLGS
LLAFAVYTILKWLEYHNANPGQDDDNFVKKTPAGFNIPTGDRQYSNSTNGAKEHTPSEPRDSGVVQTNSGPQGFQ
AV*
>Trichoderma_harzianum_485859
MAKPLRKFTERFKTALVIGLGEFCGTFMFLLLSFMGAQAALDNGPDGGKLDASTLLYIASSFGTALAVNVWVFYR
VTGGMFNPAVTLGLVLVGAVKPLRALIIILPMQIAAGIAAAATVSGLLPGPLSVTNSLGSGTSIVQGLFIEMFLTA
QLVLTVYFLAVEKHRATFLAPVGIGVSVFIAHMAGTNFTGTGINPVRSLGPAVVTGSFRGYHWIYWLGPCLGALL
SFTVYSLLKGLEYQIANPGQDAGDPETANEEAIAAAEMGQEGLAMVRSRSRSMSVPNDMMFKKEREAAMRRASVP
EESPMSAADVV*

>Trichoderma_arundinaceum_8551
MDVAAFDGSFAPLVRPQAVRLTPWYRRRDYFVGQWTDVSLWKSAVVELVATSCLVFLSGQITATLESYGTPQVGG
YIGISNIILISTFIYATAPASGGHLNPMITFSAVLTGLCSVPRGILYMAGQTLGGALAGGILLGVWGRQRATSLQ
GGGCWYDPSQASSGQIYLNEVFASFVLLFLSFGVGLDPRQAAAFGPRMGPLLVGTSLGLVSFATSGIIPGYAGAQ
MNPTRCLAFGIARRDMSYQWVYWFGPAVGSIMMGILYNLIPPHHTELSKKKSKKVTDSMAEHTEVPLV*
>Trichoderma_asperellum_64497
MALATFDGSFAPLVRPQDVRLTPWYRRKDYYMGQWFEPALWRSAIVELIATCCQVFVSGQIAATIQSYGTPQIGA
YIGISNLVMISTFIYAVAPASGGHINPTITFASVLTGLCPVPRGILYMIGQTAGGALAGGILLGIWGEERAKAVH
GGGCWYDPSQANPGQIYLNETFASFVLLFLAFGVGLDPRQAALFGPRLGPVLVGASLGLVSFSTSGIIPGYAGAQ
MNPAKCLGNGIARLDLSYQWIYWFGPAVGGIMMGIFYNLIPPHHVELCKLKSREMSRQITSDSMAERAEAPVVGT
V*
>Trichoderma_atrobrunneum_88171
MDLAAFDGSFAPGVRPGAVRLTPWYRRRDYFVGQWADISVWKSAVVEFVATSCLVFLSGQITATLESYQTPQVGG
YIGISNIILIATFIYATAPASGGHLNPMITFSAILTGLCSVPRGILYMCAQTLGGALAGGILLGVWGPKRATSLQ
GGGCWYDPSQANPGQIYLNEVFASFVLLFLSFGVGLDPRQAALFGPRMGPLLVGASLGLVSFATSGIIPGYAGAQ
MNPSRCLAFGIARRDMTYQWVWWFGPAVGCLMMGVFYNLIPPHHVELSKEKSRESRLNSMAGNTEVPEV*
>Trichoderma_brevicompactum_122570
MDLAAFDGSFAPLVRPRVVRLTPWYRRKDYFVGEWTDLSLWKSAVVELVATSCLVFVSGQITATLESYGTPQVGA
YIGISNIILISTFIYATAPASGGHLNPMITFSAVLTGLCPVPRGILYMAGQTLGGALAGGILLGVWGPQRATSLQ
GGGCWYDPSQATPGQIYLNEVFASFVLLFLSFGVGLDPRQAALFGPRMGPLLVGTSLGLVSFATSGIIPGYAGAQ
MNPTRCFAFGIARRDMSYQWVYWFGPAVGSIMMGILYNLIPPHHTELAKKKSKEVNSDSMAEHTEVPLV*
>Trichoderma_citrinoviride_1108082
MDLATFDGSFAPAVRPRGVRLTPWYRSRDYFVGQWLDVSVWKSAVVEMIATSCLVFISGQITATIEGYGTPQVGG
YIGISNIILLSTFIYATAPASGGHLNPLITFSAILTGLCSVPRGILYLSGQTLGGALAGGLLLGVWGRERATSLQ

GGGCWYDPSQASPGQVYLNEAFSSFVLLFLSFGVGLDPRQAALFGPRMGPLLVGASLGLVTFSSSGIIPGYAGAQ
MNPSRCLAYGIARRNMTYQWVWWFGPAVGALMMAVFYNLIPPHHTELTKQKSKETEPDTMAGHTDIPVV*
>Trichoderma_gamsii_JPDN01000001.1
MDLAAFDGSFAPLVRPQAVRLTPWYRRRDYFVGQWFEPALWRSAVVEMIATCCQVFVSGQIVATISTYGTPQLGA
YIGISNLVLIATFIYAVAPASGGHMNPMITFAAVLTGLCSVPRGILYMVGQTAGGALGGGILLGIWGKERAIAVR
GGGCWYDPSQANPGQIYLNETFASFVLLFLAFGVGLDPRQAALFGPRMGPALVGASLGLVTFSTSGIIPGYAGAQ
MNPAKCFGNGIARLDLSYQWIYWFGPAVGGIMMGILYNLIPPHHAELSKRKSREMSREITSDSMAERAEASVIAS
S*
>Trichoderma_guizhouense_LVVK01000020.1
MDLAAFDGSFAPGVRPGAVRLTPWYRRRDYFVGQWVDISVWKSAVVEFVATSCLVFLSGQITATLESYGTPQVGG
YIGISNIILIATFIYATAPASGGHLNPMITFSAILTGLCSVPRGILYMCAQTLGGALAGGILLGVWGPKRATSLQ
GGGCWYDPSQANPGQIYLNEVFASFVLLFLSFGVGLDPRQAALFGPRMGPLLVGASLGLVSFATSGIIPGYAGAQ
MNPSRCLAFGIARRDMTCQWVYWFGPAVGCLMMGVFYNLIPPHHVELSKQKSKESRMNSMAGNTEVPEV*
>Trichoderma_hamatum_ANCB02000213.1
MDLAAFDGSFAPLVRPQAVRLTPWYRRRDYFVGQWFEPALWRSAIVELIATCCQVFVGGQIAATIQSYGTPQVGA
YIGISNLVMISIFIYAVAPASGGHMNPLITFAAVLTGLCSVPRGMLYIIGQTAGGALAGGILLGIWGEERAIAVR
GGGCWYDPSQANPGQIYLNETFASFVLLFLAFGVGLDPRQAALYGPRMGPVLVGASLGLVSFSTSGIIPGYAGAQ
MNPAKCFGNGIARLDLSYQWIYWFGPAVAGIMMGIFYNLIPPHHAELCKKKSREMSREITSDSMAERAEAPVVGT
V*
>Trichoderma_koningii_BCGH01000003.1
MDLATFEGSFAPAVRPRGVRLTPWYRSRDYFIGQWLDVSVWKSAVVEMVATSCLVFVSGQITATIEGYGTPQVGG
YIGISNIILLSTFIYATAPASGGHLNPMITFSAILTGLCSVPRGMVLYLCGQTLGGALAGGLLLGVWGRERATSL
QGGGCWYDPSQASPGQIYLNEVFSSFVLLFLSFGVGLDPRQAALFGPRMGPLLVGASLGLVTFSSSGIIPGYAGA
QMNPSRCLAFGIARRNMSDQWVWWFGPAVGGLIEAIVYNLIPPHHMELVKQKSKETEPDTMVGHTDIPTV*
>Trichoderma_longibrachiatum_1343482
MDLATFEGSFAPAVRPRGVRLTPWYRSRDYFIGQWLDVSVWKSAVVEMVATSCLVFVSGQITATIEGYGTPQVGG
YIGISNIILLSTFIYATAPASGGHLNPMITFSAILTGLCSVPRGVLYLCGQTLGGALAGGLLLGVWGRERATSLQ
GGGCWYDPSQASPGQIYLNEVFSSFVLLFLSFGVGLDPRQAALFGPRMGPLLVGASLGLVTFSSSGIIPGYAGAQ
MNPSRCLAFGIARRNMSYQWVWWFGPAVGGLIEAIVYNLIPPHHMELVKQKSKETEPDTMVGHTDIPTV*
>Trichoderma_parareesei_3
MDLAAFDGSFAPALRPREVRLAPWYRSRDYFVGQWLDVSVWKSAVVEMVATSCLVFLSGQITATIEGYGTPQVGG
YIGISNIILLSTFIYATAPASGGHLNPMITFSAILTGLCSVPRGILYMSAQTLGGALAGGLLLGVWGRERATSLQ
GGGCWYDPSQASPGQVYLNEVFSSFVLLFLSFGVGLDPRQAALFGPRMGPLLVGASLGLVTFSSSGIIPGYAGAQ
MNPSRCLAFGIARRNLSYQWVWWFGPAVGGLIEALLYNLIPPHHTELVKKQSIGNDPDTMVGHTDIPTV*
>Trichoderma_pleuroti_MDJU01000245.1
MDLAAFDGSFAPGVRPGAVRLTPWYRRRDYFVGQWADMSVWKSAVVEFVATSCVVFLSGQIAATLEGYGTPQVGG
YIGISNIILIATFIYATAPASGGHLNPMITFSAILTGLCSVPRGILYMCGQTLGGALAGGILLGVWGPKRATSLQ
GGGCWYDPSQANPGQIYLNEVFASFVLLFLSFGVGLDPRQAALFGPRMGPLLVGASLGLVSFATSGIIPGYAGAQ
MNPSRCLAFGIARRDMSCQWVWWFGPAVGGLMMGVFYKLIPPHHVELSKQKSKESRLNSMAGNTEVPEV*
>Trichoderma_reesei_128546
MDLAAFDGSFAPAVRPREVRLAPWYRSRDYFVGQWLDVSVWKSAVVEMVATSCLVFLSGQITATIEGYGTPQVGG
YIGISNIILLSTFIYATAPASGGHLNPMITFSAILTGLCSVPRGILYMSAQTLGGALAGGLLLGVWGHERATSLQ
GGGCWYDPSQASPGQVYLNEVFSSFVLLFLSFGVGLDPRQAALFGPRMGPLLVGASLGLVTFSSSGIIPGYAGAQ
MNPSRCLAFGIARRNMSYQWVWWFGPAVGGLIEALLYNLIPPHHTELVKKQGIGNDPDTMVGHTDIPTV*
>Trichoderma_virens_8824
MDLAAFDGSFAPGVRPHAVRLTPWYRSRDYYIGQWLDLSVWKSAVVEFIATCCMVFLSGQITATLESYGTPQVGG
YIGISNIILLSTFIYATAPASGGHLNPMISFSAILTGLCSVPRGILYMCGQTLGGALAGGILLGVWGPERATSLK
GGGCWYDPSQANPGQIYLNEVFASFVLLFLSFGVGLDPRQAALFGPRMGPLLVGASLGLVSFSTSGIIPGYAGAQ
MNPSRCLAFGIARQNMAYQWIWWFGPAVGGLMMGVLYNLIPPHHTELSKQKSKESHSSSIVGHTEIPTV*

***>Aspergillus_terreus_*AAJN01000055.1**
MVVDYLPEYEVEEHHSPLQVAIPPFAGRVGGNQDFIVDRNDPRNTKVLEKVPDAAPHMTLAEVFDFRGFLSADFW
KWAMLECVASMMNVFITAWVTTHPPAPVTAPKTEVGIYHTLTFFSPLFGGITNLLLTPLLIYTFSPSSGGHISPT
ITMATFFARIISFPRMILYLAGQTFGGALAGLALNSAYGTREFTVGGCYIDTELVPVKDALIIEFMACLILIFLA
FGVALDPRQAKIFGHATSPWFVGIVLGIVSWGTAWTRPGYIGASVNPARCFGVYVASHFPGYHWIHWIGPLAAAI
AHGVVYFIDPLWKDPSSSSSDSGRS*
***>Penicillium_marneffei_*XP_002149425.1**
MEPRSPPPDNEMGDTKIALPGRYESPITGALPAVQPFAGRIGGNQSLVLDRNDPKNSDYLKAVPDAAPFMRISEA
LDLRGFLDLNLWKFAIVEGVASFLLIFITGWIAIQPKPTSSSSASTAASSAGVFGTASFLGPLVGGITNWFFLTL
FIYCFAPVSGGHINPTITLATFFARLISFPRMVLYLIGQTAGGALAGLVLKDVYGSSDFAVGGCLVETNLVEVRQ

ALVLEFMCTLILIFLAFGVALNPRQERIYGPALAPWLVGLALGLLSWGSGYEKPGYAGASMNPARCFGVYVGSGF
PGYHWIHWVGVICATLGHGVFYQLLPPWISEKAK*
