# Supplementary material for: Fungal X-Intrinsic Protein Aquaporin from Trichoderma atroviride: Structural and Functional Considerations
Source: Biomolecules. 2021 Feb 23;11(2):338. doi: 10.3390/biom11020338 (PMC7927018; doi:10.3390/biom11020338)
Supplement: Supplementary file 1 [file biomolecules-11-00338-s001.zip › Figures Sup PDF/FigS2_detailled_TrichoMIP_alignment.pdf]

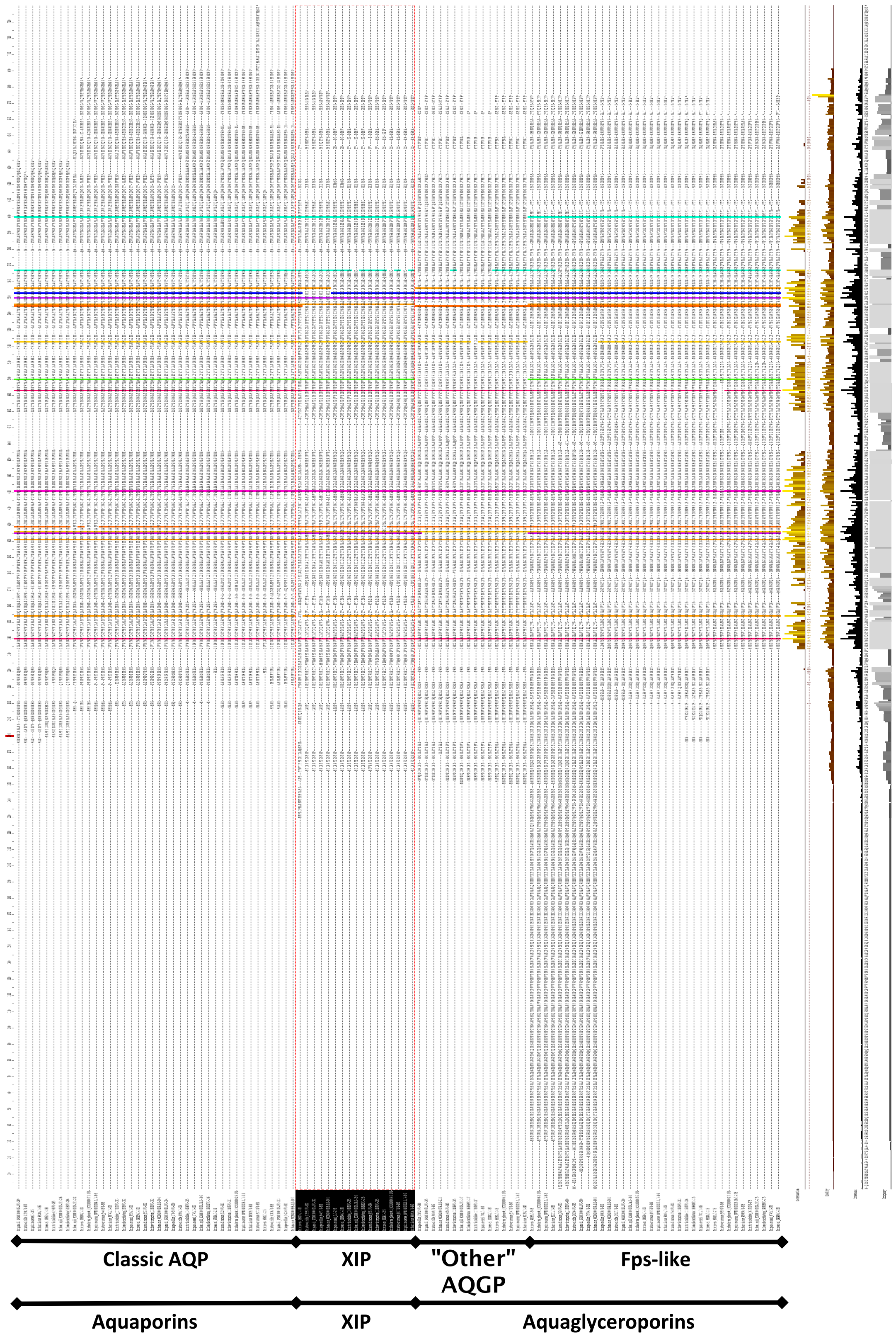

Figure S2. Multiple sequence alignment of MIP from *Trichoderma* genus. AQP, aquaporin; XIP, X-Intrinsic Protein; AQGP, Aquaglyceroporins. The XIP subgroup is highlighted by a red square. Alignments were computed with ClustalW, and the result was viewed in Jalview. Coloured residues (Taylor color code) correspond to a percentage identity threshold of 85% of identity between all sequences. This supplemental figure is provided for illustrative purposes, and the alignment can be easily read in Figure S3.
