# Supplementary material for: Fungal X-Intrinsic Protein Aquaporin from Trichoderma atroviride: Structural and Functional Considerations
Source: Biomolecules. 2021 Feb 23;11(2):338. doi: 10.3390/biom11020338 (PMC7927018; doi:10.3390/biom11020338)
Supplement: Supplementary file 1 [file biomolecules-11-00338-s001.zip › Figures Sup PDF/FigS4_TriatMIP_alignment.pdf]

```
                              10        20        30        40        50        60        70        80        90       100
TriatXIP_319992       ----------------------------------------------------------------------------------------------------
TriatAQP_6990         ----------------------------------------------------------------------------------------------------
TriatAQP_43816        ----------------------------------------------------------------------------------------------------
TriatAQP_31598        ----------------------------------------------------------------------------------------------------
TriatFpsAQGP_283564   MCHEAIGGSLWEDRLPHAGIDETISSSRQNVRGSQEDTERGSLRRRSRTSRRSTFRSVAPLTTAGQGTQFNLAGPSESQQLRSAHEPFVHPGYGDLNPSY
TriatFpsAQGP_39327    ----------------------------------------------------------------------------------------------------
TriatO'AQGP_90169     ----------------------------------------------------------------------------------------------------

                             110       120       130       140       150       160       170       180       190       200
TriatXIP_319992       ----------------------------------------------------------------------------------------------------
TriatAQP_6990         ----------------------------------------------------------------------------------------------------
TriatAQP_43816        ----------------------------------------------------------------------------------------------------
TriatAQP_31598        ----------------------------------------------------------------------------------------------------
TriatFpsAQGP_283564   EQPNNTKPIWSLAKPLPRVVRPGMVPTKNELLESCINAELPAENSQKLGLDVDPNELEKGRIDKSADVRKMAAQVTDARVQRENNFIKTILASDAESASG
TriatFpsAQGP_39327    ----------------------------------------------------------------------------------------------------
TriatO'AQGP_90169     ----------------------------------------------------------------------------------------------------

                                                                                          Cytosolic Nter extension
                             210       220       230       240       250       260       270       280       290       300
TriatXIP_319992       -----------------------------------------------------------------------------MDLAAFDGSFAPLVRPQAVRLTP
TriatAQP_6990         ----------------------------------------------------------------------------------------------MRRPSA
TriatAQP_43816        --------------------------------------------------------------------------------------------MPANRIHT
TriatAQP_31598        -----------------------------------------------------------------------------MDSLRIFKQRSVSGDNNRNRDRF
TriatFpsAQGP_283564   DVAQLVQTRSSQRRATITRPPVQSPLDTVEESVSDHLSTASKKRRSRESQRLDEAEGEIDLGPDELIEGNRSLETLRIDQEAYPEDLHPLVQNLVEDEIH
TriatFpsAQGP_39327    ----------------------------------------------------------------------------------------------MSPSLE
TriatO'AQGP_90169     ---------------------------------------------------------MTTRELNRLHTHDLELGTTEATQKHVPRNVVP-PRRISQRRLD

                                 TMH1                  LoopA        TMH2          LoopB  "NPA1"     TMH3
                             310       320       330       340       350       360       370       380       390       400
TriatXIP_319992       WYRRRDYFVGQWFEPALWRS-AVVELIATCCQVFVSGQIVATISTYGTPQLGAYIGISNLVLIATFIYAVAPASGGHMNPTITFAAVLTGLCSVPRGLLY
TriatAQP_6990         SRDTVRNELVVVFGEFYGTFMFLL-MSYIGTQAAIDNNSPGNPEAPLFPFSLMYIAASFGTALAVNVWVFYRVTGGLFNPAVTLGLVLVRAITPLRGLLV
TriatAQP_43816        LSKRYEKALVIGVGEFCGTFMFLL-LSFMGAQVALENN-PAADGHKLEPATLLYIASSFGTALAVNVWVFYRVTGGMFNPAVTLGLVLVGAVKPLRALII
TriatAQP_31598        HPSTIQKHLIASAGEFVGTFFFLW-FAYAGSMQYIKQATLSPPSGGISDTTVFFIAHVYSFSLLVNVWAFYRISGGLFNPAVTLGMCLAGTLPWVRAAFL
TriatFpsAQGP_283564   NNHTVWSVVRTHHREALAESLAVF--IQLTVG--FCADIAVTVANAGNPNT---TAWAWGFASMMGIYISGGVSGAHILNPTITVMLWFFRGFPKRKMPEY
TriatFpsAQGP_39327    EEQLAWSRIREYCMDAFSEFFGTM--VLILFGDGVVAQVVLSGSTKGDYQS---ISWGWGIAVMFGVFV-GGKSGGHILNPAVTFANCLYRGHPWRKLPVY
TriatO'AQGP_90169     FEHRRPRWLRECIAEATGVFMYVLPGIGAIATFTLNAASPIGASAFGSLFA---IGFAFALGIAFAIITCAPTSGGHFSPAVTICLCIWQGFPLKKVPYY
                                                                              H2                     NPA1

                          TMH3                    LoopC                                      LoopD      TMH5
                             410       420       430       440       450       460       470       480       490       500
TriatXIP_319992       LVGQTAGGALGGGILLGIWGKE------------RAIAVRGGGCWYDPSQANPGQIYLNETFASFVLLFLAFGVGLDPRQAALFGPRMGPALVGASLGLV
TriatAQP_6990         FPTQIVAGIAAAAVTDALL---------------------PGPLLVANK-LSSGTSISRGLFIEMFLTAQLVITVYFLAVEK----HRATFLAPLGIGLA
TriatAQP_43816        LPTQIIAGIAAAATVSALL---------------------PGHLDVTNS-LGGGTSTAQGLFIEMFLTAQLVLTVYFLAVEK----HRATFLAPVGIGVS
TriatAQP_31598        VPAQIIASMCAGGLARCMF---------------------PGDLAVANSVLSSDTSIVRGLFIEMFFTAFLVFVVLMLSAER----SKDTFIAPIGIGLA
TriatFpsAQGP_283564   FLAQFIGAFCACFAAYGVYYVSIQHYVSS---G--TGTDQEIMNCFVTNQRNPYINVPTALFTEFVGTMCLTVVVLALGDDQ--NAPPGAGMNSLIIGLV
TriatFpsAQGP_39327    ALAQLLGAMVGAAIVYGNYKSAFDAFEGG--AGIRTVTGPTASAGVFCTYPAPFMTRTGMFFSEFIASSILMFCIFALVDPN--NVNAGH-MMPLALFFL
TriatO'AQGP_90169     IFSQLVGGFLAALVLMGIYHQQINEMKELLLAAGKPLVANGAPASILCSFPNPDQSMGYVFFTEFICDCFVGFIIWAAIDPA--NPFISPAVAPLMIGLA

                        TMH5  LoopE  "NPA2"           TMH6                        Cytosolic Cter extension
                             510       520       530       540       550       560       570       580       590       600
TriatXIP_319992       SFSTSGIIPGYAGAQMNPAKCFGNGIARMDL-SYQWIYWFGPAVAGIMMGILYNLIPPHHAELCKRKSREMSREMTSDSMAERAEASVIASA--------
TriatAQP_6990         VFIAHICGTNFTGTGINPARSFGPCVVTS-FTGYQWIYWAGPFMGALLAFAVYSILKWLEYHNANPGQDDDSNVKRNPAGFAITSNDRQYSS---STTGS
TriatAQP_43816        VFIAHLAGTNFTGTGINPVRSLGPAVVTGRFEGYHWIYWLGPCLGALLSFAVYSLLKALEYQIANPGQDAGDPETANEEALAVEEMGTEGLAIVRSRTRS
TriatAQP_31598        LFVAMLAGTSYTGASLNPVRSFGCAVATPSFPGYEWIYWLGPFMGALVAAGFYRFVKLSHYEEANPNREETDHPTGDQP---------------------
TriatFpsAQGP_283564   IVCLTISFANQTGAALNPSRDFGPRLALLAL-GYGSELFT-NPYWFYGPFAGTLMGSFTGAFLYDFFIFTGGESPVNYPLDRTQRALRKSGMKWRRRLHL
TriatFpsAQGP_39327    IFGIGACFGWETGYAINLARDFGPRLVSYMI-GYGHEVWSAGGYYFWIPMVAPFCGTAFGGFLYDVFLYTGN-SPINTPMLGLTRLFRPRKNVWS---NT
TriatO'AQGP_90169     YAAMAWGFGDVT-LTMNMARDLGPRIVAAI--FFGKEAFSYKNYSPIAILVSIPATMVSSAFYEFVFRDSVSVIQSGHAVHEDGDEALVRHITRTTTVEG
                         H2         LE1  LE2
                                      NPA2

                             610       620       630
TriatXIP_319992       --------------------------------
TriatAQP_6990         KPHTPTGHNPRD----SGIAQTNSAPQGFQAV
TriatAQP_43816        GSAASEARYKRDVAMRRGSAVDGSAVSAADAV
TriatAQP_31598        --------------------------------
TriatFpsAQGP_283564   TPKEEEDKVV----------------------
TriatFpsAQGP_39327    RPSAIDTKV-----------------------
TriatO'AQGP_90169     IDERRGDYKS----------------------
```

Figure S4. Aminoacid sequence alignment of the seven MIP from *Trichoderma atroviride*. The highest residue similarity between MIP members are shown in red (100%) and green (>80%). The six transmembrane helices (TMH1-6) and the two hemi-helicoidal regions ("NPA1" and "NPA2") which include the "NPA" motives "NPA1" (in Loop B) and "NPA2" (in Loop E) (framed in red) are shown with grey highlights. The colors used for the schematic representation of the 8 TMH are related to Figure S6. The four amino acid residues constituting the ar/R constriction region (H1-H2-LE1-LE2) are shown with blue highlights.
