# Supplementary material for: Fungal X-Intrinsic Protein Aquaporin from Trichoderma atroviride: Structural and Functional Considerations
Source: Biomolecules. 2021 Feb 23;11(2):338. doi: 10.3390/biom11020338 (PMC7927018; doi:10.3390/biom11020338)
Supplement: Supplementary file 1 [file biomolecules-11-00338-s001.zip › Figures Sup PDF/FigS5_XIP_trichoderma_alignment.pdf]

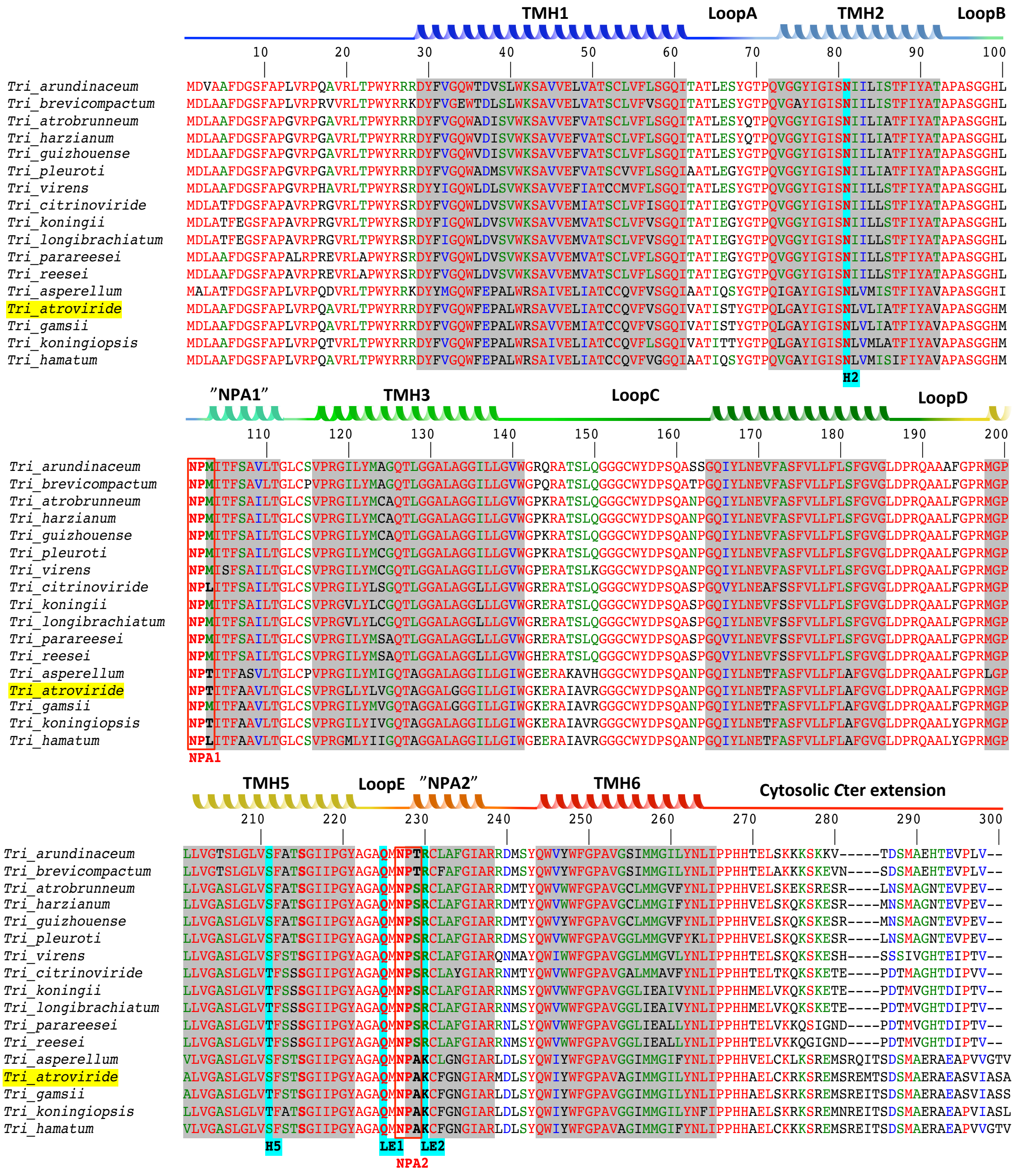

Figure S5. Aminoacid sequence alignment of the fungal XIP from *Trichoderma* genus. The highest residue similarity between XIP members are shown in red (100%) and green (>80%). The six transmembrane helices (TMH1-6) and the two hemi-helicoidal regions ("NPA1" and "NPA2") which include the "NPA" motives "NPA1" (in Loop B) and "NPA2" (in Loop E) (framed in red) are shown with grey highlights. The colors used for the schematic representation of the 8 TMH are related to Figure S6. The four amino acid residues constituting the ar/R constriction region (H1-H2-LE1-LE2) are shown with blue highlights.
