# Supplementary material for: Fungal X-Intrinsic Protein Aquaporin from Trichoderma atroviride: Structural and Functional Considerations
Source: Biomolecules. 2021 Feb 23;11(2):338. doi: 10.3390/biom11020338 (PMC7927018; doi:10.3390/biom11020338)
Supplement: Supplementary file 1 [file biomolecules-11-00338-s001.zip › Figures Sup PDF/FigS6_TriatMIP_3D.pdf]

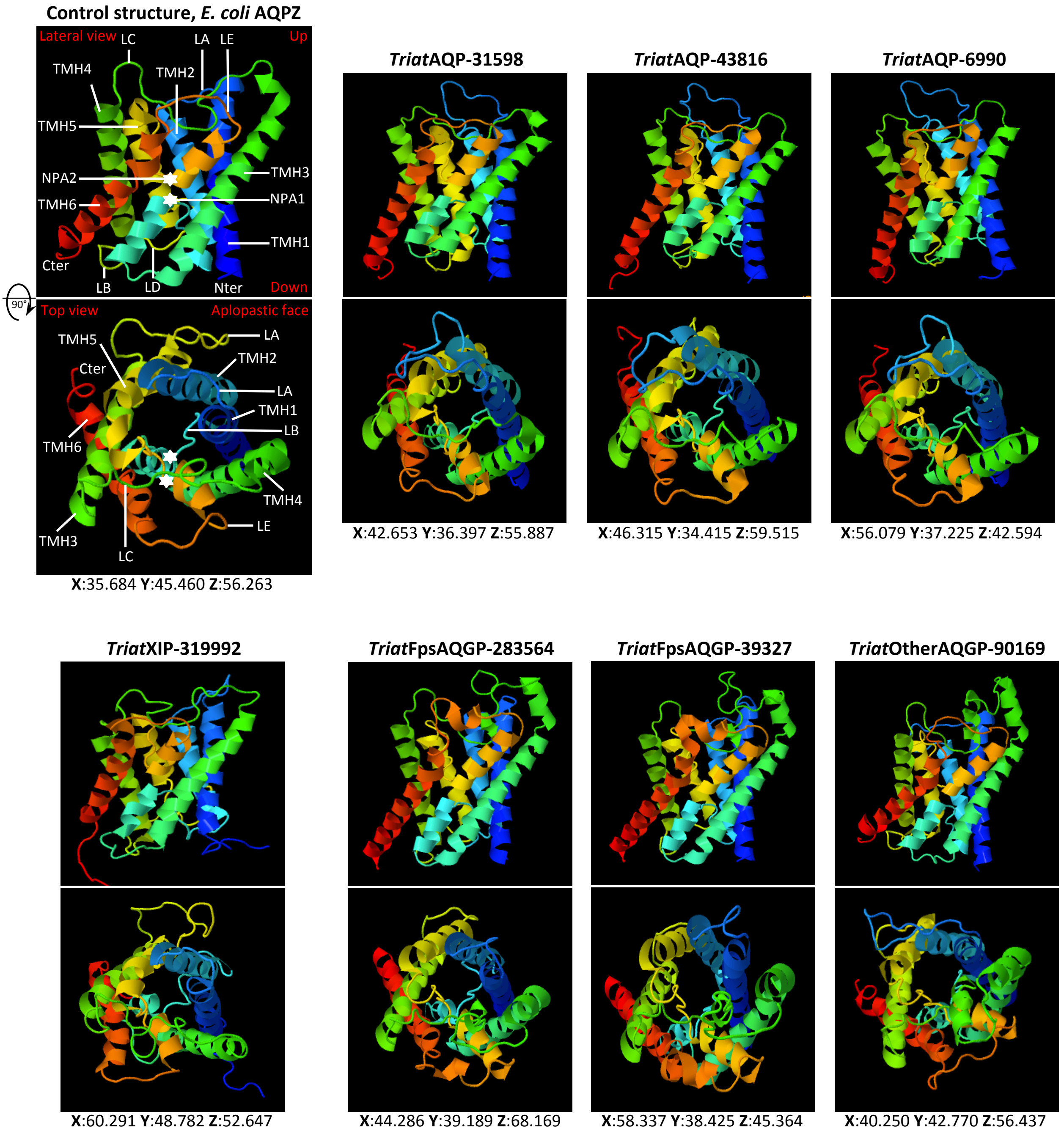

Figure S6. Predicted 3D models of the seven *Trichoderma atroviride* MIP proteins. Models were generated by using the PHYRE2.0 Protein Fold Recognition server, using the Normal mode modeling based on alignment to experimentally solved protein structures. Because the cytoplamic *N*ter and *C*ter extensions do not feature potential secondary structures, they were manually truncated ten amino acids upstream and down of the TMH1 and TMH6 for analyses, respectively. Each member is represented in both lateral (top structure related to the apoplastic side) and top views (apoplastic side). The modeling of *Trichoderma atroviride* aquaporins *Triat*AQP-31598, *Triat*AQP-43819, *Triat*AQP-6990 was carried out by comparison with the c2w2eA template. The fungal XIP *Triat*XIP-319992 was modeled with C6pojA. The aquaglyceroporins *Triat*FpsAQGP-283564 and *Triat*FpsAQGP-239327, and the Other-Aquaglyceroporin *Triat*OtherAQGP-90169 were modeled with C6f7hA, c6n1gA and d1fx8a templates, respectively. A 97% minimum of residues from each *Triat*MIP sequence was modeled at 100% confidence level. Models were visualized by rainbow color from N to C terminus, where the intracellular (B,D) and extracellular (A,C,E) loops and transmembrane helices (TMH1 in dark blue, TMH2 in light blue, TMH3 in dark green, TMH4 in light green, TMH5 in yellow, TMH6 in red) are shown, as illustrated with the control structure (AQPZ, Aquaporin-like from *Escherichia coli;* d1fx8a). Cytosolic *Cter* and *N*ter extensions were truncated for the analysis. X, Y and Z model dimensions were expressed in Angström.
