# Supplementary material for: Fungal X-Intrinsic Protein Aquaporin from Trichoderma atroviride: Structural and Functional Considerations
Source: Biomolecules. 2021 Feb 23;11(2):338. doi: 10.3390/biom11020338 (PMC7927018; doi:10.3390/biom11020338)
Supplement: Supplementary file 1 [file biomolecules-11-00338-s001.zip › Figures Sup PDF/FigS7_TriatMIP_cell_localisation.pdf]

*TriatXIP*_319992	**Plas** (22), E.R. (3), Mito (1)
*TriatAQP*_6990	**Plas** (13), Mito (2), E.R. (2), Vacu (1)
*TriatAQP*_31598	**Plas** (24), Pero (1)
*TriatAQP*_43816	**Plas** (15), Vacu (5), Mito (4), E.R. (2)
*TriatO'AQGP*_90169	**Plas** (27)
*TriatFpsAQGP*_283564	**Plas** (21), E.R. (2), Cyto (1), Pero (1), Golg (1)
*TriatFpsAQGP*_39327	**Plas** (23), E.R. (3)

Figure S7. Details of predicted subcellular localization of *Trichoderma atroviride* MIP. Predictions were made by using Wolfpsort server. Abbreviations of protein localization sites in the dataset are as follows: Plas, plasma membrane; E.R., Endoplasmic reticulum; Mito, mitochondria; Vacu, Vacuolar; Pero, peroxisomes. The number mentioned against localization roughly indicates the number of nearest neighbors to the query which localizes to each site, but are adjusted to account for the possibility of dual localization. The strongest occurrences are highlighted in bold.
