# Supplementary material for: Fungal X-Intrinsic Protein Aquaporin from Trichoderma atroviride: Structural and Functional Considerations
Source: Biomolecules. 2021 Feb 23;11(2):338. doi: 10.3390/biom11020338 (PMC7927018; doi:10.3390/biom11020338)
Supplement: Supplementary file 1 [file biomolecules-11-00338-s001.zip › Figures Sup PDF/FigS8_RMSD_TriatXIP.pdf]

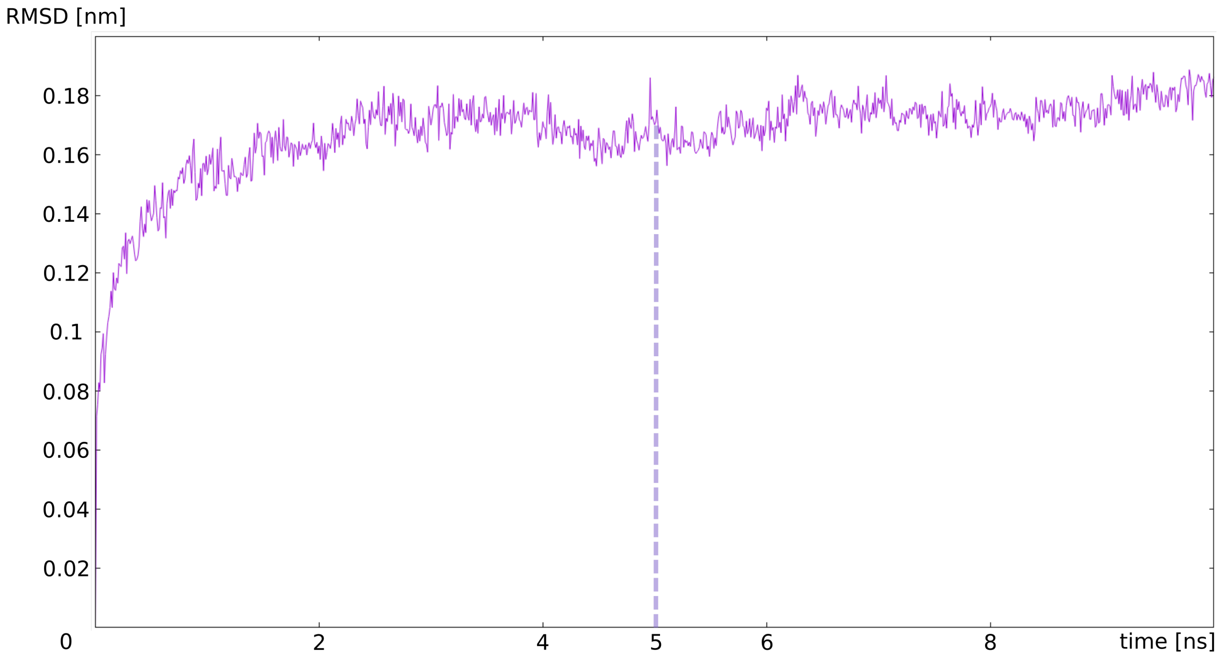

Figure S8. Root mean square displacement of the alpha carbons of the tetrameric modeled structure of *Triat*XIP (without the extra-cellular loops and intra-cellular extremities) along the whole 10 nanoseconds production trajectory. The reference structure corresponds to the structure at time t = 0. For all the analyses, only the last 5 nanoseconds were used as they corresponded to a stabilized plateau phase.
