# Supplementary material for: Fungal X-Intrinsic Protein Aquaporin from Trichoderma atroviride: Structural and Functional Considerations
Source: Biomolecules. 2021 Feb 23;11(2):338. doi: 10.3390/biom11020338 (PMC7927018; doi:10.3390/biom11020338)
Supplement: Supplementary file 1 [file biomolecules-11-00338-s001.zip › Figures Sup PDF/TableS1_Primers.pdf]

Table S1. Primers used for the *TriatXIP* mutant construction, and the *Trichoderma atroviride MIP* qPCR amplifications

Primers used for the *TriatXIP* mutant construction

**XIP5' end**	5'-flank region (1,05 kb to ATG)	GGCCGGCAGGAATGATGGGCCG
**XIP3' end**	3'-flank region	CTTCCAACCTTCACGCCGATGC

Primers used for the *TriatXIP* mutant validation

**XIP5' end**	Including the start codon	GAAAGCATGGAGCCGCTACAAGAG
**XIP3' end**	Including the stop codon	CTTCCAACCTTCACGCCGATGC

Primers used for *q*PCR amplification

	**Accession number [JGI]**	**Primer sequences [5' to 3' ; Forward / Reverse]**
**18SrDNA**	18SrDNA	GGTGGAGTGATTTGTCTG / CTTACTAGGGATTCCTCG
**28SrDNA**	29846	TTTGAGTAAGAGCATACGGGGCC / GTTGATACATTCGAATGCCCACGT
**Actin**	297070	AAGGACCTCTACGGCAACATTG / GACAATGGAGGGACCGCTC
**Tubuline**	307163	AGGTCAACCGTGCTGTCTGCATG / CAAACTTAGTACTCAGCCTCCATC
**GPdH**	143663	CCAGAACATCATCCCCAGCAGC / GATGGAAGAGTTGTTGTTGCCGAG
**Aquaporin**	31598	GCATCACTCAATCCAGTTCG / CTGTGCTGGTTATGGCTGGTCG
	43816	GGCGAATGAGGAGGCGCTGG / GACGCTCAAACAGCATCAGCC
	6990	GTGTCGTCACCAGCTTCACG / CTCTTGACGTTGCTATCGTCG
**Aquaglyceroporin**	39327	CCATGGTAGCCCCATTCTGCG / GGTGGCAGCCATTAGACTTTGG
	283564	GCTCATGGGCTCATTCACCG / CTAAACAACCTTGTCTTCTTCC
	90169	GAACATGGCTCGAGACCTGGG / GAACATGGCTCGAGACCTGGG
**X-Intrinsic Protein**	319992	CAGTGGATTTACTGGTTTGGGC / CTTCACGCCGATGCAATCACCG
